# Editorial: Reviews in: Radiopharmaceuticals in nuclear medicine

**DOI:** 10.3389/fmed.2023.1178528

**Published:** 2023-03-15

**Authors:** Chuen-Yen Lau, Mostafa Yuness Abdelfatah Mostafa

**Affiliations:** ^1^HIV Dynamics and Replication Program, Center for Cancer Research, National Cancer Institute, National Institutes of Health, Bethesda, MD, United States; ^2^Department of Physics, Faculty of Science, Minia University, Minya, Egypt

**Keywords:** radiopharmaceutical, nuclear medicine, molecular imaging, radionuclide therapy, tracer

## Radiopharmaceuticals are ever evolving

Medical application of radiopharmaceuticals began in the 1920s. The first diagnostic procedure utilizing a radiotracer was performed with ^214^Bi, then known as radium C, to measure arm-to-arm blood transit time (1, 2). Applications are far more sophisticated now, with nuclear medicine currently using radiopharmaceuticals for both clinical care and research evaluations. For example, ^18^F-Fluorodeoxyglucose (FDG)-positron emission tomography (PET) is commonly used to detect metabolically active cancer lesions; it is also being evaluated for ability to detect Human Immunodeficiency Virus (HIV) reservoirs (3). In addition to diagnostic uses, radiopharmaceuticals can be employed therapeutically or as theranostics, which combine diagnostic with therapeutic functions that share a specific target (4). This collection of reviews of radiopharmaceuticals in nuclear medicine (https://www.frontiersin.org/research-topics/38645/reviews-in-radiopharmaceuticals-in-nuclear-medicine) highlights ongoing innovation in the field.

## Key messages from the collection

Two articles in the collection address PET imaging for lung cancer. Martucci et al. have performed a meta-analysis to quantitatively assess the impact of ^18^F-FDG PET/Computed tomography (CT) on small cell lung cancer (SCLC) staging. This builds on prior evidence, which relied primarily on ^18^F-FDG PET without concordant anatomical imaging. Based on 6 publications, the meta-analysis indicates that ^18^F-FDG PET/CT changes binary stage (limited vs. extensive disease) in approximately 15% of cases when compared to conventional staging. Since clinical management depends on the disease stage, incorporation of ^18^F-FDG PET/CT into SCLC staging has the potential to improve outcomes. Prospective studies will be helpful to validate this finding and to assess actual impact on outcomes such as morbidity and mortality.

As the armamentarium of radiopharmaceuticals is continuously expanding, Zhu et al. provide a helpful review of PET tracers for lung cancer beyond ^18^F-FDG. These alternative radioligands target tumor markers and cellular function, including proliferation, amino acid metabolism and transport, tumor hypoxia, angiogenesis, tyrosine kinases and cancer-associated fibroblasts. Many of these tracers are still under evaluation. As the more promising candidates progress to clinical and human research applications, they present the opportunity to target specific features of lung cancer and personalize patient management. Furthermore, the rational approach to ligand discovery, based on understanding of lung cancer pathogenesis, can be applied to diverse other conditions.

Identifying and understanding CNS disease *via* molecular imaging has long been a goal of the field. As exemplified by the pursuit of radiotracers for Alzheimer's Disease, for which targets include β-amyloid plaques, TAU tangles, and neurodegeneration (5), diverse targets must be explored based on the current understanding of disease features. Tong et al. offer an overview of Poly (ADP-ribose) polymerases (PARPs) as radioligand targets in central nervous system (CNS) diseases. Building on recent studies demonstrating PARP1 activation in neurodegenerative conditions and aging (6), the authors delve into how the PARP1 pathway might be targeted for diagnostic and therapeutic purposes. The development of PARP1 radiotracers, which are being evaluated in clinical trials for CNS conditions as well as malignancies, will hopefully lead to new insights in efforts to mitigate CNS disease. A major question will be how and if they contribute to improved outcomes.

The last article in the collection focuses on radionuclide therapy for metastatic or otherwise inoperable paragangliomas and pheochromocytomas (Prado-Wohlwend et al.). Radionuclide therapy serves as an alternative to chemotherapy and other targeted medications currently under investigation. Using modeling, Prado-Wohlwend et al. found that ^177^Lu-Lu-DOTA-TATE resulted in better progression free survival when compared to ^131^I-metaiodobenzylguanidine (MBG). Progression free survival with ^177^Lu-Lu-DOTA-TATE improved with proportion of adrenal lesions and pheochromocytomas. Understanding the impact of different therapies on disease course, particularly when treatment options are limited, is critical and should be validated with prospective clinical studies.

## Building on current knowledge

While these reviews demonstrate incredible progress in nuclear medicine over the past several decades, they also highlight the great potential for further advancement. Identification of novel disease-specific ligands will engender greater clinical relevance and support more personalized use of radiopharmaceuticals. Researchers should strive to optimize the impact on disease course by using these agents to elucidate pathogenesis and identify novel opportunities for intervention. The development of such probes must consider disease attributes, current treatment and management approaches, and target characteristics. For results stemming from use of diagnostic radioligands, potential ethical concerns about whether knowledge it provides regarding a condition is actionable must be considered when seeking full licensure of the agent.

Ensuring that radioligands can access protected sites, particularly the CNS will also be necessary. In addition to optimizing the size and isoelectric point of a candidate, modifications can be made to use carriers or metabolic pathways that actively accumulate the ligand at the desired site. Another burgeoning arm of nuclear medicine is targeting of specific pathogens. For example, progress made in imaging tuberculosis and HIV must be continued (7–9). A particular challenge will be imaging of diffuse, low-concentration targets as is the case with certain infections.

## Conclusions

**Figure 1:**
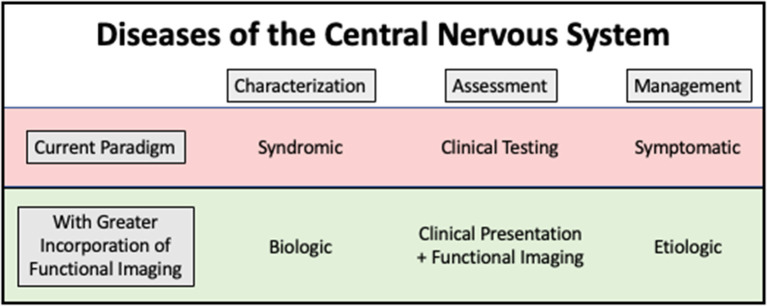
Conceptual model showing how functional imaging has the potential to change our approach to treatment and monitoring of central nervous system diseases. By using functional imaging to understand biology and pathogenesis, underlying causes of disease can be more effectively addressed.

Molecular imaging contributes significantly to research on and clinical management of many conditions. It also has the potential for an even greater impact. Figure 1 shows how functional imaging could impact clinical management, using the example of CNS diseases (10).

Many conclusions derived from modeling, retrospective, or observational data need prospective high-quality clinical studies to validate results and guide clinical use. For example, we need additional data to conclude that use of ^18^F-FDG PET/CT for staging improves morbidity and mortality from SCLC. Continued development of additional disease and infection-specific ligands should also be supported, with the goal to personalize management more effectively, improve morbidity and mortality, and optimize quality of life. Radiopharmaceuticals have a very bright future (11). Ongoing assessment and building on current evidence, as has been done by the articles in this collection, will facilitate continued progress.

## Author contributions

All authors contributed to conceptualization, writing, and approval of the final version of the manuscript.
